# The fifth Japanese meeting on biological function and evolution through interactions between hosts and transposable elements

**DOI:** 10.1186/s13100-022-00261-7

**Published:** 2022-01-13

**Authors:** Kenji Ichiyanagi, Kuniaki Saito

**Affiliations:** 1 Laboratory of Genome and Epigenome Dynamics, Department of Animal Sciences, Graduate School of Bioagricultural Sciences, Nagoya University, Furo-cho, Chikusa-ku, Nagoya, 464-8601 Japan; 2 Invertebrate Genetics Laboratory, National Institute of Genetics, Yata 1111, Mishima, Shizuoka, 411-8540 Japan

**Keywords:** Transposable elements, Retrotransposition, Evolution, Epigenetics, RNA

## Abstract

The fifth Japanese meeting on host–transposon interactions, titled “Biological Function and Evolution through Interactions between Hosts and Transposable Elements (TEs),” was held online on August 26–27, 2021. The meeting was supported by National Institute of Genetics and aimed to bring together researchers studying the diverse roles of TEs in genome function and evolution, as well as host defense systems against TE mobility by chromatin and RNA modifications and protein-protein interactions. Here, we present the highlights of the talks.

## Introduction

Transposable elements (TEs), which stay and/or move around the genome, constitute a substantial part of the eukaryotic genome. Some of them play key biological and evolutionary roles. The interactions between TEs and their hosts were discussed in a series of meetings titled “Biological Function and Evolution through Interactions between Hosts and Transposable Elements” [1, 2], held at the National Institute of Genetics (NIG), Mishima, Japan. These meetings allowed TE researchers working in Japan to gather and exchange ideas. At the fifth meeting, held online on August 26–27, 2021, we invited 18 speakers to give talks over seven sessions, which were chaired by Takuji Tsukiyama (Kindai University, Japan), Yoichi Shinkai (RIKEN, Japan), Akihiko Koga (Kyoto University, Japan), Kensuke Kataoka (National Institute of Basic Biology, Japan), Kuniaki Saito (NIG, Japan), Fumitoshi Ishino (Tokyo Medical and Dental University, Japan), and Hidenori Nishihara (Tokyo Institute of Technology, Japan). The meeting had approximately 100 attendees, many of whom contributed to constructive discussions throughout the sessions.

## Highlights of the talks

### Regulation of TEs at the transcriptional and post-transcriptional levels

In general, DNA methylation and histone H3 K9 trimethylation (H3K9me3) play pivotal roles in the inhibition of transcription of TEs in plants, fungi, and animals. Hidetaka Ito (Hokkaido University) explained how plant retrotransposons are regulated by DNA methylation in multiple ways. Using a heat-inducible retrotransposon *ONSEN* as a model, his group first discovered that *ONSEN* transcription was reduced rather than increased in DNA methyltransferase mutants. Therefore, unlike other TEs, DNA methylation appeared to be required for *ONSEN* to drive its transcription. However, they subsequently revealed in a DNA methyltransferase mutant that the localization of other DNA methyltransferases was altered to compensate for *ONSEN* methylation [3]. This phenomenon suggested the presence of a TE repression mechanism in the host plant, where multiple DNA methyltransferase(s) could work as a backup when the primary enzyme responsible for DNA methylation is no longer functioning. This shed a new light on the struggle between TEs and their hosts and provided new insights into their survival strategies.

Tetsuji Kakutani (University of Tokyo), one of the organizers, has been working on the silencing and anti-silencing of TEs. In *Arabidopsis* mutants defective in DNA methylation, diverse TEs were mobilized, demonstrating the importance of DNA methylation for defense against the proliferation of TEs [4]. Interestingly, *VANDAL21*, one of the TEs immobilized by DNA methylation, had the ability to counteract the defense via the protein VANC21 encoded in the element. When VANC21 was expressed from a transgene, endogenous *VANDLA21* lost DNA methylation and was mobilized [5]. The target sequences of VANC21 and related proteins had evolved rapidly through the formation of tandem repeats [6]. Yusaku Tanaka (University of Tokyo) from the Kakutani lab reported the unpublished crystal structure of the VANC21 protein, which highlighted the coevolution of VANC proteins and their target sequences.

In animal germ cells, H3K9me3, DNA methylation, and piRNAs are major players in TE silencing, and these mechanisms interact with each other. In fruit flies, both germ and somatic cells utilize piRNAs to repress TE transcription through the H3K9me3 deposition. Keita Miyoshi (NIG, Japan) described his unpublished results on the Sov protein (CG14438), a TE silencing effector mediated by the piRNA pathway in *Drosophila*. Using the *Drosophila* ovarian somatic cell line (OSC), he investigated whether Sov regulates the TE-related chromatin status. Knockdown of Sov increased the level of H3K4me2 without affecting the level of H3K9me3, suggesting the involvement of an H3K9me3-independent mechanism, whereby the acquisition of H3K4me2 at TE sites to drive transcription was inhibited by Sov. Masaki Kawase (Nagoya University, Japan) presented unpublished results using germ-cell-specific knockout mice for *Setdb1*, which encodes a H3K9 methyltransferase. In spermatocytes of *Setdb1*-deficient mice, several members of the ERVK family were shown to be derepressed, and caspase-mediated apoptosis was induced. Comparison of transcriptomes and epigenomes of *Setdb1*- and *Dnmt3l*-deficient spermatocytes [7] further revealed that H3K9me3 and DNA methylation differentially regulated the ERVK and L1 families, respectively.

Repressive modifications, such as H3K9 and DNA methylation, are often associated with heterochromatinization, and this process requires the heterochromatin protein HP1. Makoto Tachibana (Osaka University, Japan) presented recent studies showing that HP1 proteins not only formed compact chromatin, but also stabilized the H3K9 methyltransferases. He generated mouse embryonic stem cells lacking all HP1 paralogs and found that HP1-depletion induced protein degradation of H3K9 methyltransferases, Suv39h1, Suv39h2, Setdb1, and the G9a/GLP complex. Chase analysis indicated that Suv39h1 and Setdb1 mutants with impaired HP1 interaction degraded faster than their respective wild-types. Moreover, an HP1 mutant protein that could bind to the methyltransferases but not to H3 methylated at K9 was not able to stabilize the methyltransferases. These results suggested that HP1 might protect H3K9 methyltransferases from protein degradation by anchoring them to heterochromatinic compartments in the nucleus.

To reveal the mechanism for heterochromatin establishment, Atsuko Shirai (RIKEN, Japan) used an ectopic heterochromatin monitoring system in which an EGFP gene fused to centromeric (alphoid DNA in humans) or pericentromeric (major satellite in mice) repeats were inserted into a specific AAVS locus in HEK293 cells with the VloxP-SloxM1 site-specific recombination. In this system, EGFP expression was shut off when the neighboring repeats form heterochromatin. As expected, EGFP fluorescence diminished approximately 20 days after the repetitive DNA insertion into the locus. ChIP analysis showed the accumulation of H3K9me3 in the EGFP region. DNA methylation was also observed. These results suggested that heterochromatin can be formed after ectopic insertion of (peri) centromeric repeats, and the ongoing molecular processes can be monitored for further study.

Recent studies have indicated that post-transcriptional modification of mRNA is involved in the regulation of the expression of TE-encoded proteins. One of the most abundant chemical modifications in RNA is N6-methyladenosine (m6A), which is found in both coding and non-coding RNAs, including TEs. Yuka Kabayama (University of Edinburgh, UK) presented an unpublished study on the potential mechanisms for RNA degradation via m6A and its reader protein YTHDF2. Several residues of YTHDF2 were found to be phosphorylated in adult mouse testes, which could impede the binding of YTHDF2 to m6A or recruit factors such as the CCR4-NOT complex to initiate deadenylation of mRNA.

Genetic studies have indicated that TEs use host factors for their mobility [8, 9], although the details remain unclear. Mammalian LINE-1 (L1) encodes two proteins, ORF1p and ORF2p, which are necessary for their mobility. Both bind to the L1 RNA to form a ribonucleoprotein (RNP) complex prior to L1 retrotransposition, and ORF2p also has endonuclease and reverse transcriptase activities in vitro. Tomoichiro Miyoshi (Kyoto University, Japan) studied the host proteins that restrict or facilitate L1 retrotransposition, and previously revealed the interaction between L1 ORF2p and DNA repair proteins, such as PARP2 and RPA, that facilitated L1 retrotransposition [10]. In this meeting, he presented unpublished work demonstrating the interactions between a network of interferon-stimulated gene (ISG) proteins and L1 RNPs. However, unlike PARP2 and RPA, these ISG proteins inhibited L1 retrotransposition through different mechanisms, suggesting multifaceted interactions between L1 and the host.

It is also important to understand the host conditions that induce TE mobility. Kazuya Iwamoto (Kumamoto University, Japan) previously reported increased L1 copy number in schizophrenia brain cells and animal models of schizophrenia [11]. The poly(I:C) model was used in the animal model, where a dsRNA analog was injected into pregnant mice. In this meeting, he presented that the epigenetic states of the promoters of specific L1 loci were altered upon poly(I:C) injection. These data were obtained from the subfamily specific analysis they developed [12]. He also introduced new tools for the L1 study, including the integrated experimental scheme for single-cell retrotransposition mapping, bioinformatics pipeline to analyze full-length L1 loci using next-generation sequencing data, and newly-developed retrotransposition monitor mice. These tools will be released to the community and are expected to contribute to a deeper understanding of the L1 role in the brain.

Tomoyuki Honda (Okayama University, Japan) reported correlations between human L1 retrotransposition and tumorigenesis. His group found that L1 retrotransposition was increased during the infection process of an oncogenic virus, Kaposi’s sarcoma-associated herpesvirus (KSHV) [13]. Upon infection, a KSHV-encoded gene, vFLIP, activated L1 retrotransposition through ICAM upregulation and subsequently downregulated MOV10, a host protein that inhibits L1 retrotransposition reactions. Interestingly, inhibition of L1 expression could suppress vFLIP-mediated transformation, suggesting that enhanced L1 retrotransposition was involved in KSHV-induced tumorigenesis. On the other hand, the group found that the retrotransposition activity of an intact L1 in the naked-mole rat (NMR) genome, named NMR-L1, was extremely low [14]. This strong repression of NMR-L1 activity might be implicated in the pronounced cancer resistance observed in NMRs. Together, their studies led to the consideration that L1 retrotransposition may promote tumorigenesis by introducing deleterious oncogenic mutations and inducing genomic instability [15].

### Involvement of TEs in the host evolution

Taisei Kikuchi (University of Miyazaki, Japan) discussed TEs in *Caenorhabditis inopinata,* a sibling species of *C. elegans. C. inopinata* was recently discovered in Okinawa, Japan by his team, and showed striking morphological and ecological differences from *C. elegans,* despite their close phylogenetic relationship. Genome analyses revealed that *C. inopinata* had > 10 times more TEs in its 120 Mb genome than *C. elegans* [16]. They revealed that *C. inopinata* lacked certain key genes in the small endogenous RNA pathways, which might have allowed TEs to expand within the genome. Moreover, the population genome analysis of wild strains as well as the mutational analysis of laboratory strains revealed a high degree of TE mobility in its genome, making *C. inopinata* an attractive research model to investigate TE regulation in nematodes and their contributions to morphological and/or ecological evolution.

Individual insertion events of TEs are potential drivers of phenotypic diversification. After illustrating that severe acute respiratory syndrome coronavirus 2 (SARS-CoV-2) had caused higher rates of morbidity and mortality in Europe than in East Asia, Yasuo Ariumi (Kumamoto University, Japan) showed that the severity of coronavirus disease 2019 (COVID-19) was genetically linked to an insertion of Alu, a member of the short interspersed elements (SINEs), in the angiotensin converting enzyme 1 (ACE1) gene. His group found an insertional polymorphism of a 287-bp Alu copy in the 16th intron of the human ACE1 gene, which might reduce the gene expression. Interestingly, there was a significant negative correlation between the frequency of the inserted allele in a population and the number of SARS-CoV-2 cases and the number of deaths due to SARS-CoV-2 infection [17]. Thus, this Alu insertion in ACE1 appeared to cause a variation in the host resistance to SARS-CoV-2, and the difference in the genotype distribution at this site among ethnic groups may explain the apparent difference in mortality between the West and East Asia [18].

Introns of animal and plant genomes often accumulate many TEs [19], although such TEs can induce heterochromatic environments in these genic regions. Hidetoshi Saze (Okinawa Institute of Science and Technology, Japan) presented his recent study on intronic TEs and their epigenetic regulation in *Oryza sativa* rice [20]. In the rice genome, intronic TEs were generally shorter than intergenic TEs. TEs tend to accumulate in promoter-proximal introns, which are associated with repressive heterochromatin modifications, such as DNA methylation and histone H3K9 methylation. Interestingly, most genes containing heterochromatic TEs were transcriptionally active and showed tissue-specific or stress-responsive expression patterns. This study suggested that intronic TEs may have an impact on gene regulation and genome evolution in plants.

Intergenic TE insertions can also affect the host epigenome and transcriptome. Kenji Ichiyanagi (Nagoya University, Japan) presented his recent work showing that copies of the mouse B2 SINE comprised a boundary of DNA methylation and histone modifications by both CTCF-dependent and -independent mechanisms. Comparison of two mouse subspecies revealed that B2 retrotransposition had created > 100 CTCF binding sites and modulated the expression of nearby genes over the past million years [21]. He also introduced a new method, termed melRNA-seq, that allowed accurate SINE expression analysis at a single-locus level [22]. He and his colleagues revealed that more than 3000 SINE loci were transcriptionally active in spermatogenic cells, but their expression levels varied by three orders of magnitude with a power-law distribution.

## Concluding remarks

In this meeting, we discussed various topics with regards of TE-host interactions at the molecular level within a wide range of organisms, especially in terms of the transcriptional and post-transcriptional regulation of TE mobility. Recent technical advances, such as long-read sequencing, have contributed to genomics studies on TE biology. In particular, analyses of individual TE loci rather than family-level analysis for expression, chromatin modification, de novo transposition site, etc., are now feasible and will elucidate the contributions of individual TE insertions to host evolution. On the other hand, it is also interesting to study whether accumulated TE copies in bulk would contribute to the regulation of epigenetic landscape and/or higher-order chromatin configurations. The authors expect to see some advances in these fields in the next meeting, which will be held in 2023.

## Data Availability

not applicable.

## Abbreviations

AAVS: Adeno-associated virus integration site
ACE1: Angiotensin converting enzyme 1
ChIP: Chromatin immunoprecipitation
COVID-19: Coronavirus disease 2019
ERVK: Endogenous retrovirus K
H3K9: lysine-9 of histone H3
ISG: Interferon-stimulated gene
KSHV: Kaposi’s sarcoma-associated herpesvirus
L1: LINE-1
LINE: Long interspersed element
NIG: National Institute of Genetics
NMR: Naked mole-rat
ORF: Open reading frame
OSC: Ovarian somatic cell
RNP: Ribonucleoprotein
SARS-CoV-2: Severe acute respiratory syndrome coronavirus 2
SINE: Short interspersed element
TE: Transposable element
